# Preparation of Photocurable Organic–Inorganic Hybrid Composites for Continuous Manufacturing of 3D-Patterned Abrasive

**DOI:** 10.3390/ma17163977

**Published:** 2024-08-10

**Authors:** Kitae Kim, Jaehan Jung

**Affiliations:** 1 Department of Materials Science and Engineering, Hongik University, Sejong-si 30016, Republic of Korea; 2 Department of Materials Science and Engineering, Seoul National University of Science and Technology, Seoul 01811, Republic of Korea

**Keywords:** surface modification, photocurable, hybrids, roll-to-roll, 3D pattern

## Abstract

Photocurable hybrid organic–inorganic composites were prepared via surface modification and 3D-patterned structures were fabricated by utilizing a continuous roll-to-roll manufacturing strategy. The surfaces of nanocrystals were engineered with a bifunctional ligand that is a 2-carboxyethyl acrylate, which possesses a carboxylic acid moiety at one end and an acrylate functionality moiety at the other end, yielding acrylate-functionalized nanocrystals. Micro-scale 3D patterns (protruding pyramidal shapes with each side measuring 147 μm) were continuously manufactured at a speed of 2.5 m/min via UV curing with a soft engraved mold. The surface properties of the functionalized nanocrystals and their UV curing condition were explored with Fourier transform infrared spectroscopy. The morphology of the 3D film was measured using scanning electron microscopy. A pin-on-disk tribometer measurement revealed an improved interaction between the functionalized particles and resins.

## 1. Introduction

Abrasives are used in a wide range of industries, including automotive, machinery, and electronics [1]. Various abrasive techniques, such as slurry lapping, grinding wheels, and grits on a belt have been developed. Among these techniques, abrasives with fixed grits on a belt backing are an attractive option due to their improved surface finish and reduced slurry waste disposal [2,3,4]. Fused or sintered particles such as Al_2_O_3_, SiC, and ZrO_2_ are typically used as abrasive grains due to their excellent mechanical properties and cost-effectiveness [5,6]. These grits are fixed using vitreous, organic, or metallic bonds that retain the abrasive grain and control the wear rate. Traditionally, coated abrasives were fabricated by simply applying grits onto the belt backing using thermosetting resins. For example, grits were attached to the belt backing using either phenolic or polyimide thermosetting resins with plasticizers. However, such a manufacturing process inevitably results in uncontrolled orientation of the grits and irregular shapes, thereby leading to the uneven grinding of substrates [7].

In this context, a microreplication technique has been utilized to fabricate abrasives with controlled shapes, such as protruding pyramidal abrasives, enabling the production of microscale well-aligned 3D-patterned structures [8,9,10]. Such a well-controlled structure (e.g., protruding pyramidal structure) provides channels for the efficient removal of lapping swarf, thereby enhancing cutting ability by supplying a continuous stream of fresh abrasive mineral. In early studies, protruding pyramidal abrasives were manufactured through the hot filament chemical vapor deposition of diamond onto a silicon wafer [11]. Specifically, the silicon master underwent oxidation, lithography, and etching to create a template, after which pyramidal-shaped diamonds were grown by CVD using hydrogen and methane gas at 900 °C. The combination of the protruding architecture and the exceptional mechanical properties of diamond offers significant abrasive capacity, durability, and a prolonged lifetime. Despite the excellent morphological quality of the resultant products, the chemical vapor deposition process is not cost-effective and is time-consuming, making it unsuitable for large-scale fabrication [12,13,14]. In this context, cost-effective soft-lithography approaches, utilizing soft molds such as PDMS and urethane to produce patterned structures, can be applicable in manufacturing abrasives [15,16,17,18].

Herein, we introduce a scalable continuous manufacturing method for creating micro-scale patterned films using a roll-to-roll approach. The continuous manufacturing process for fabricating 3D-patterned film via roll-to-roll involves a two-step procedure. Initially, a seamless engraved urethane mold is prepared utilizing a micro-patterned copper roll. Simultaneously, a patterned hybrid composite film is continuously UV-cured by capitalizing on the prepared soft engraved urethane mold. It is worth noting that this process allows for continuous production without risking damage to the costly micro-patterned metal roll. UV-curable functional hybrid composites for durable abrasives are synthesized by introducing photo-curable functionalities onto nanocrystal (NC) surfaces through surface modification. This approach utilizes a bifunctional surfactant, 2-carboxyethyl acrylate, which possesses a carboxylic acid for binding with NCs on one end and an acrylate moiety for UV curing on the other end. The UV curing condition is explored using Fourier transform infrared (FTIR) spectroscopy. The morphology of the resulting patterned hybrid film is characterized using scanning electron microscopy (SEM). Additionally, a pin-on-disk tribometer measurement is performed to demonstrate the enhanced interaction between the functionalized particles and resins.

## 2. Materials and Methods

All chemicals, including alumina, 2-carboxyethyl acrylate (CEA), poly(ethylene glycol) diacrylate (PEGDA), and diphenyl(2,4,6-trimethylbenzoyl)phosphine oxide (TPO) from Sigma Aldrich (St. Louis, MO, USA); KBr, methanol, and *N*-methylformamide (NMF) from Alfa Aesar (Haverhill, MA, USA); and alumina powder; and polyethylene terephthalate (PET) film from Eungwang industries (Seoul, Republic of Korea), were used as received.

*Surface modification*: To modify the interfacial property of alumina particles, CEA was attached onto their surface via ligand exchange. Specifically, 200 mg of alumina powders were dispersed in 5 mL of CEA and they were refluxed at 45 °C for 1 day. They were then purified with methanol three times and dried in a vacuum oven.

*3D pattern fabrication*: To continuously produce engraved soft molds, a copper metal roll with a diameter of 360 mm and a length of 1 m was employed. The metal roll featured microsized pyramidal patterns. An acrylate urethane oligomer and a 2 wt% TPO mixture were used to produce a urethane engraved mold using an embossed copper metal roll. Specifically, acrylate urethane monomers, 2 wt% of photo-initiator, and TPO were vortex-mixed for 2 h. This mixture was then applied between a patterned copper roll and a 125 μm thick PET film, followed by photo-curing using 365 nm UV irradiation at an intensity of 5000 mJ/cm^2^. The resulting urethane mold on the PET substrate was continuously produced with a rolling speed of 2.5 m/min. It is worth noting that the PET film was continuously supplied. Subsequently, a 50 wt% particle blend, comprising either CEA-functionalized alumina or pristine alumina, was vortex-mixed with 2 wt% of TPO and PEGDA. This blend was then poured onto the urethane mold on the conveyor belt, with a 125 μm thick PET film attached. The final step involved UV irradiation at 365 nm, resulting in photo-polymerization and subsequent detachment of the final product from the hydrophobic urethane mold, yielding a 3D-patterned hybrid film. For the continuous mass production of films, the conveyor belt speed and light intensity were optimized. The UV irradiation intensity was varied from 5000 mJ/cm^2^ to 15,170 mJ/cm^2^, and the fabrication speed was controlled at 1 m/min, 2.5 m/min, and 5 m/min.

*Characterization*: The morphology of the 3D-patterned films was studied by SEM (Hitachi S-4800, Hitachi-shi, Japan). The FTIR spectra were taken by a Bruker alpha II (Berlin, Germany) spectrometer. To prepare the FTIR samples, a volume of 4 μL of the mixture solution was dropped on KBr pellets. To test the photopolymerization degree of the mixture, the solution on KBr pellets was exposed to UV light at 365 nm with an intensity of 5000 mJ/cm^2^ for 0 s to 60 s. A pin-on-disk tribometer was used to measure the capping capability of the CEA surfactant with alumina. In detail, abrasive films fabricated with CEA–alumina and pristine alumina were placed on a disk with a loading of 5 N at 160 rpm using a 6 mm track diameter. The pin was made of a SUJ2 ball with a radius of 6 mm, and the disk was rotated for 1 h.

## 3. Results and Discussion

**Figure 1:**
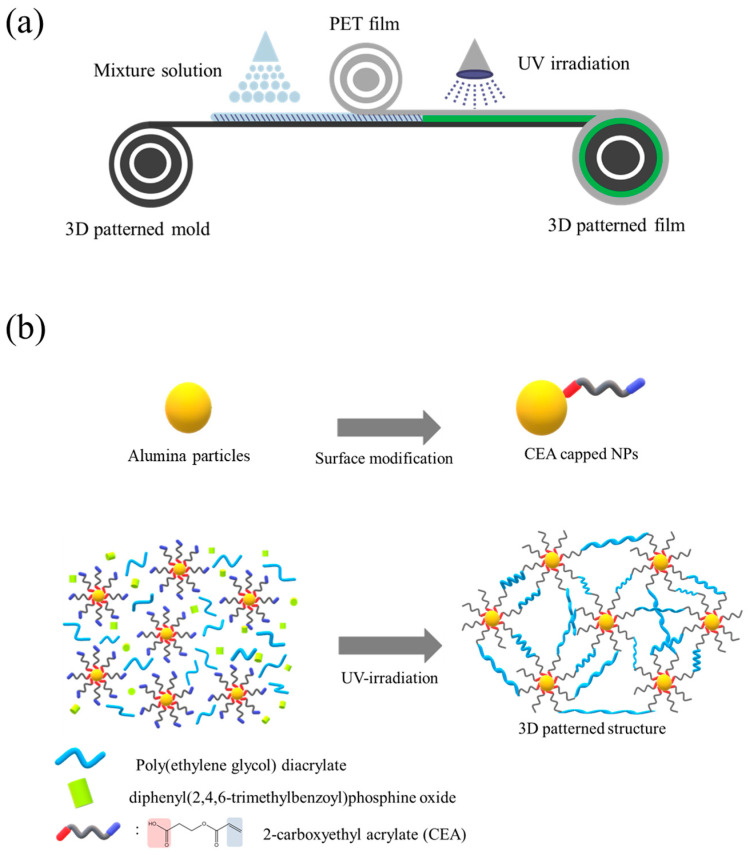
Schematic illustrations of (**a**) the continuous fabrication of the 3D-patterned films and (**b**) the synthetic route to hybrid patterns by capitalizing on the surface engineering and UV irradiation.

The continuous fabrication of 3D-patterned organic–inorganic hybrid nanocomposite films via photo-polymerization is shown in Figure 1a. It utilized advantageous continuous roll-to-roll processing, which is suitable for mass production. Specifically, a patterned urethane engraved mold was fabricated through photo-polymerization using an embossed micro-patterned copper roll (depicted on the left side of Figure 1a). Subsequently, the organic–inorganic hybrid mixture was poured onto the prepared engraved urethane substrate, and a PET film was placed on top of the mixture. Finally, the composites were cured by UV irradiation, and the final product was detached from the urethane mold, resulting in a hybrid 3D-patterned film. It is worth noting that urethane engraved mold produced via roll-to-roll processing with a patterned metal roll ensured continuous manufacturing of the 3D-patterned film without risking damage to the expensive patterned metal mold [19].

To achieve excellent dispersion of inorganic particles within the organic photocurable resin (i.e., acrylate resin), the surfaces of the nanoparticles were modified as depicted in Figure 1b. This involved ligand exchange with a bifunctional surfactant that was a 2-carboxyethyl acrylate (CEA), which possess a carboxylic acid functionality for binding with particles at one end and an acrylate moiety for photopolymerization at the other end [20]. Acrylate-functionalized alumina particles were then dispersed in photocurable resins such as poly(ethylene glycol) diacrylate (PEGDA), and CEA, as depicted in the lower row of Figure 1b. These hybrid photo-curable composites were utilized in subsequent fabrication steps.

**Figure 2:**
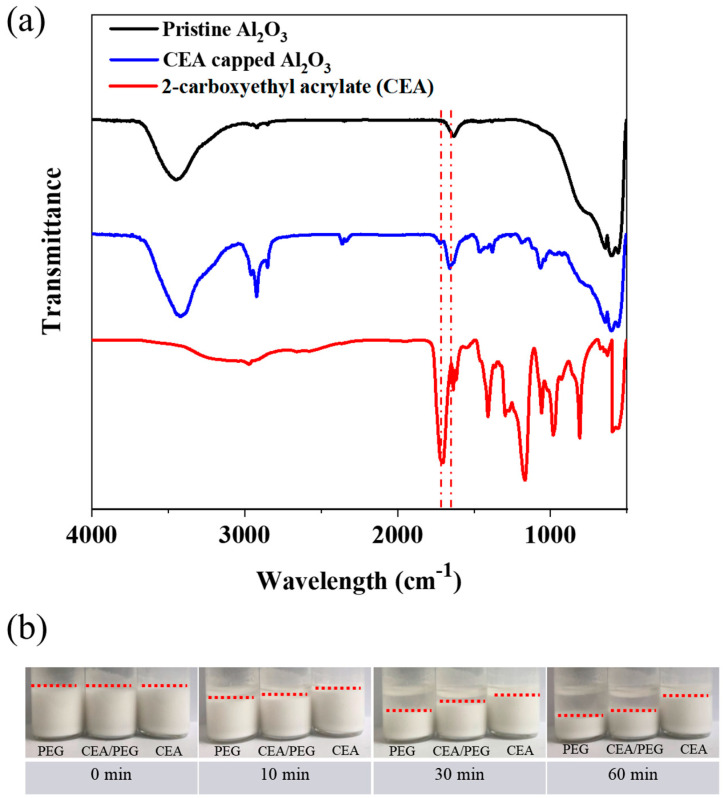
(**a**) FTIR spectra of alumina, CEA-functionalized alumina, and CEA. (**b**) Digital images of plain alumina particles dispersed in PEGDA, CEA-alumina in PEGDA, and CEA-alumina in CEA.

The occurrence of the surface modification of alumina was substantiated with FTIR, as shown in Figure 2a. The spectrum of CEA-functionalized alumina (blue) was compared with those of CEA (red) and pristine alumina (black). Absorption signals at 1721 and 1187 cm^−1^ corresponding to C=O and C-O stretching from CEA were clearly observed (blue), indicating the successful surface passivation of alumina with CEA [21,22]. The influence of surface properties on the dispersion of alumina particles in photocurable resins (e.g., PEGDA and CEA) was investigated. The digital images in Figure 2b represent plain alumina NCs dispersed in PEG, CEA-NCs in PEG, and CEA-NCs in CEA, respectively. The degree of their precipitation was monitored over 1 h, with the weight fraction of the alumina powders set at 40 wt% for all samples. Not surprisingly, CEA-functionalized NCs showed superior dispersion in PEG compared to their counterparts (plain NCs in PEG). CEA-capped NCs in CEA demonstrated the most excellent dispersibility.

**Figure 3:**
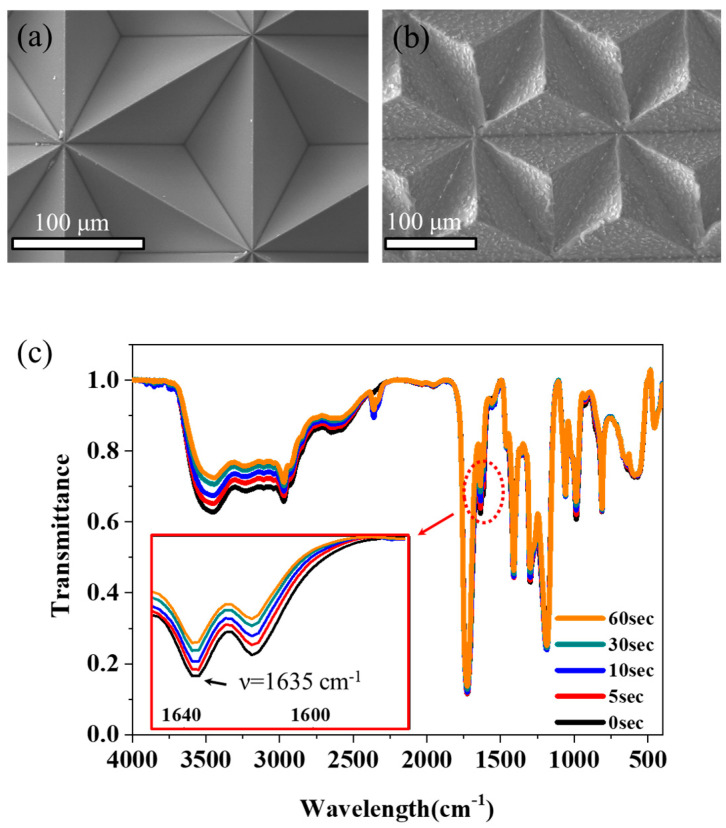
SEM images of (**a**) urethane mold and (**b**) 3D-patterned films fabricated with CEA-functionalized alumina in CEA/PEGDA. (**c**) FTIR spectra of CEA-capped alumina particles in PEGDA for UV irradiation times of 0, 5, 10, 30, and 60 s.

The 3D-patterned organic–inorganic hybrid film, composed of CEA-capped alumina and PEG resin, was fabricated on PET substrates using a urethane engraved mold via photopolymerization. The urethane mold, continuously produced using an embossed metal roll (as depicted in the left part of Figure 1a), features engraved pyramidal patterns with each side measuring 147 μm, as characterized by SEM (Figure 3a). Figure 3b presents an SEM image of the final product (i.e., 3D-patterned film). This image clearly shows protruding pyramidal shapes with each side measuring 147 μm, which is consistent with the dimensions of the urethane mold. The photopolymerization degree of the film was determined by exploring the FTIR spectra. Figure 3c presents the FTIR spectra of the organic–inorganic hybrid film for UV irradiation times ranging from 0 s to 60 s. It is evident that the absorption intensity at 1635 cm^−1^, attributed to the acrylate moieties of the CEA and PEGDA resins, decreases with increasing UV exposure time, indicating the formation of the film via photopolymerization [23,24]. This suggests that the acrylate moieties were sufficient to induce polymerization. 

**Figure 4:**
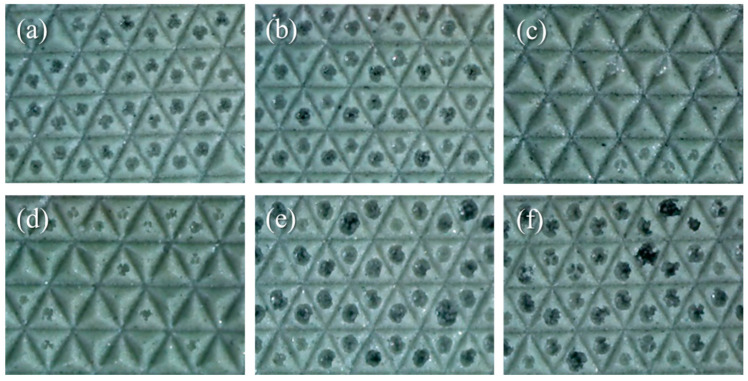
Optical images of patterned films fabricated using UV intensities of (**a**) 5000, (**b**) 6720, (**c**) 8290, (**d**) 11,960, (**e**) 14,400, and (**f**) 15,170 mJ/cm^2^.

For the continuous mass production of films, the intensity of UV irradiation was first optimized. Figure 4 presents optical microscope images of the film fabricated using UV intensities ranging from 5000 mJ/cm^2^ to 15,170 mJ/cm^2^, with a rolling speed of 2.5 m/min. It is evident that voids appeared in the vertex of the pyramid patterns when a low intensity was used. This can be attributed to insufficient irradiation energy for crosslinking. When UV intensities between 8000 and 12,000 mJ/cm^2^ were utilized, there was an observable improvement in the quality of the film. However, exceeding 12,000 mJ/cm^2^ resulted in unsuccessful patterns, where the vertexes of the pyramidal structures broke during the detachment procedure. This occurred because the vertexes had strongly polymerized with the urethane mold, causing them to adhere to the mold during detachment. 

**Figure 5:**
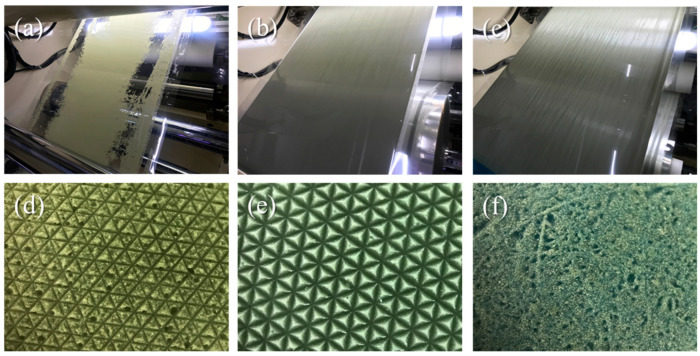
Digital images of resin coatings on PET and their patterns fabricated at rolling speeds of (**a**,**d**) 5 m/min, (**b**,**e**) 2.5 m/min, and (**c**,**f**) 1 m/min.

To optimize the roll-to-roll line speed, the production line speed was adjusted while maintaining a constant UV intensity of 8290 mJ/cm^2^. In Figure 5, digital images depict the hybrid resin coating on the PETs and their patterns after detachment. The rolling speeds were set to 1, 2.5, and 5 m/min. At a rolling speed of 1 m/min, a thick coating formed on the PET substrate. Consequently, there was insufficient UV curing energy for successful polymerization. The film morphology after detachment clearly shows unsuccessful transfer of the patterns. Conversely, a fast rolling speed of 5 m/min resulted in a thin coating, preventing pattern formation altogether.

**Figure 6:**
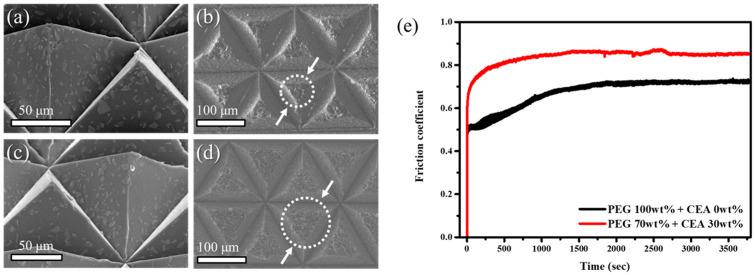
(**a**,**c**) SEM images of films fabricated using CEA-capped alumina in PEGDA and pristine alumina in PEGDA. (**b**,**d**) Worn-out images of the resulting films after a pin-on-disk tribometer test. (**e**) Friction coefficient curves of films fabricated using CEA-capped alumina in PEGDA and pristine alumina in PEGDA.

In Figure 6a,c, SEM images depict films fabricated using CEA-functionalized alumina in CEA/PEGDA and pristine alumina in PEGDA, respectively. In detail, CEA serves as both a surfactant and a photocurable resin, while diphenyl(2,4,6-trimethylbenzoyl)phosphine oxide (TPO) acts as a photo-initiator [25,26]. We mixed 50 wt% of alumina and 2 wt% of TPO with a mixture of CEA and PEGDA. The weight ratio of CEA to PEGDA was 0 wt% to 30 wt%. The film fabricated using CEA-functionalized alumina in CEA/PEGDA (Figure 6a) demonstrates superior dispersion of alumina within the matrices compared to the film fabricated using plain alumina in PEGDA (Figure 6c). This enhancement is attributed to CEA’s ability to improve the dispersion of alumina in PEGDA. It is noteworthy that the hydrophilic nature of polyether (i.e., PEGDA) facilitates the excellent dispersion of hydroxy-terminated alumina in the PEGDA matrix.

The capping capability of the CEA bifunctional surfactant with alumina was examined using a pin-on-disk tribometer instrument [27,28]. The durability of patterned abrasive films fabricated with CEA-alumina and pristine alumina was compared by rotating each film for 1 h on a disk with a loading of 5 N at 160 rpm. The extent of wear in the 3D-patterned abrasive films was assessed by determining the diameter of the worn vertex of the pyramids. Figure 6b,d represent the worn-out film fabricated using CEA-functionalized alumina in CEA/PEGDA and plain alumina in PEGDA. Interestingly, the diameter of the worn vertex of the film fabricated using CEA-alumina (Figure 6d) was approximately 46.8 μm, while it was 65.9 μm in the case of the film fabricated with pristine alumina (Figure 6b). This is not surprising, because the carboxylic acid in CEA can effectively enhance coordination with alumina particles, thereby providing improved tethering of alumina particles in the matrices upon UV curing. Figure 6e presents the friction coefficient curves for the abrasive films fabricated with CEA-alumina and pristine alumina, respectively. The friction coefficient of the abrasive film prepared by CEA-alumina shows a higher value than that of pristine alumina. This is because CEA enables excellent dispersion of alumina particles in the matrix, thus improving their abrasive ability.

## 4. Conclusions

In sum, we have introduced a large-scale, cost-effective, and continuous manufacturing roll-to-roll procedure for the fabrication of 3D-patterned organic–inorganic films using photopolymerization. Pyramidal-patterned films, with each side measuring 147 μm, were continuously manufactured at a speed of 2.5 m/min. Photocurable hybrid organic–inorganic nanocomposites composed of acrylate-functionalized NCs and acrylate resins were employed to fabricate the patterned films. Carboxylic acid-containing acrylate monomers improved the coordinate interaction with alumina particles, and hence, significantly enhanced their dispersion in matrices, as supported by FTIR and SEM characterization. A pin-on-disk tribometer measurement also demonstrated the improved interaction among alumina particles and resins. Photocurable hybrid organic–inorganic nanocomposites may serve as an important material in 3D printing industries. 

## Data Availability

The original contributions presented in the study are included in the article, further inquiries can be directed to the corresponding author.
